# Response to Letter to the Editor

**DOI:** 10.1177/20494637241264633

**Published:** 2024-07-24

**Authors:** Alex McConnachie, Alison Mitchell, Lesley Somerville, Nicola Williams, Jonathan McGhie, Gordon McGinn, Jiyoung Lee

**Affiliations:** 1 3526 University of Glasgow, Glasgow, UK; 2 3529 NHS Greater Glasgow and Clyde, Glasgow, UK

Dear Editor,

We are grateful for the interest in our article,^1^ and we agree with much of what Mercadante are say.^2^ We do not claim that IDDS results in improved survival. The article speaks only of the possibility of an association, and we are clear that we cannot infer causality from these results. Nevertheless, it is at least biologically plausible that effective pain relief in these patients could result in improved survival. We have also found that patients who successfully receive IDDS subsequently spend a greater proportion of their remaining days in the community setting.^3^ Whilst both papers suffer from biases due to deficits of retrospective data, we feel that they tell an interesting story that requires further investigation. We acknowledge the need for investments to develop large-scale prospective registries, in order that more robust analyses can be performed to better inform the treatment options for a patient group in great need of support.

We completely agree that IDDS is an effective treatment modality for a select number of patients and that it is best considered as part of a multi-professional team. The service where this study was conducted is led by Palliative Care physicians and allows for comprehensive assessment and consideration of all available treatments to enable appropriate patient selection.
